# Folate Receptor Alpha Autoantibodies in Autism Spectrum Disorders: Diagnosis, Treatment and Prevention

**DOI:** 10.3390/jpm11080710

**Published:** 2021-07-24

**Authors:** Natasha Bobrowski-Khoury, Vincent T. Ramaekers, Jeffrey M. Sequeira, Edward V. Quadros

**Affiliations:** 1 School of Graduate Studies, SUNY Downstate Medical Center, Brooklyn, NY 11203, USA; natasha.bobrowski-khoury@downstate.edu; 2 The Autism Center, University of Liège, 4000 Liège, Belgium; vramaekers@skynet.be; 3 Department of Medicine, SUNY Downstate Medical Center, Brooklyn, NY 11203, USA; jeffrey.sequeira@downstate.edu

**Keywords:** autism spectrum disorders, folate receptor alpha, folates, pregnancy, brain development, fetal development

## Abstract

Folate deficiency and folate receptor autoimmune disorder are major contributors to infertility, pregnancy related complications and abnormal fetal development including structural and functional abnormalities of the brain. Food fortification and prenatal folic acid supplementation has reduced the incidence of neural tube defect (NTD) pregnancies but is unlikely to prevent pregnancy-related complications in the presence of folate receptor autoantibodies (FRAb). In pregnancy, these autoantibodies can block folate transport to the fetus and in young children, folate transport to the brain. These antibodies are prevalent in neural tube defect pregnancies and in developmental disorders such as cerebral folate deficiency (CFD) syndrome and autism spectrum disorder (ASD). In the latter conditions, folinic acid treatment has shown clinical improvement in some of the core ASD deficits. Early testing for folate receptor autoantibodies and intervention is likely to result in a positive outcome. This review discusses the first identification of FRAb in women with a history of neural tube defect pregnancy and FRAb’s association with sub-fertility and preterm birth. Autoantibodies against folate receptor alpha (FRα) are present in about 70% of the children with a diagnosis of ASD, and a significant number of these children respond to oral folinic acid with overall improvements in speech, language and social interaction. The diagnosis of folate receptor autoimmune disorder by measuring autoantibodies against FRα in the serum provides a marker with the potential for treatment and perhaps preventing the pathologic consequences of folate receptor autoimmune disorder.

## 1. Background

Folate, an umbrella term used for metabolically active forms of folic acid (B9), is an essential B-complex vitamin necessary for basic cellular metabolism including, but not limited to, essential cellular DNA synthesis, repair and methylation including regulation of synthesis and metabolism of monoamine neurotransmitters. As a nutrient found in green leafy vegetables, legumes and fruits, it is readily absorbed by the upper small intestine after breakdown from polyglutamates to monoglutamates. Folate in its active forms facilitates one-carbon transfer reactions and contributes to the synthesis of purines, pyrimidines and amino acids [1]. One of its most characterized roles is facilitating single carbon transfer to homocysteine to form methionine. This reaction is critical for maintaining intracellular S- adenosyl methionine, an essential compound for methylation reactions. Folate also has a co-dependent relationship with vitamin B_12_ in that both vitamins must be present in adequate amounts for conversion to the physiologic forms that participate in metabolic reactions. If folate and B_12_ are not adequate, cellular metabolism and replication is interrupted [2,3]. This is most critical during fetal and neonatal development because inadequate folate during this period can result in interruptions in brain development leading to structural abnormalities that produce functional deficits of the CFD syndrome. Low cerebro-spinal fluid (CSF) folate is a characteristic feature of CFD syndrome, as first described by Ramaekers and Blau [4]. On rare occasions, CFD can also result from mutations in the FRα gene [5,6,7], but the most common cause of low CSF folate in CFD is the presence of anti-folate receptor antibodies (FRAb) that can block folate transport across the choroid plexus [8,9]. A recent report has identified mutations in the *CIC* transcription factor gene in children diagnosed with CFD syndrome. Mutations in the *CIC* gene decrease the expression of FRα to reduce folate transport across the choroid plexus [10]. No abnormalities of the FRα gene are found in ASD, but a majority of these children are positive for FRAb and have low CSF folate [11,12]. This is *a priori* proof that FRα is the primary transporter of folate into the brain under physiologic folate status.

## 2. Folate Requirements during Pregnancy

Since the discovery of its role in megaloblastic anemia and spina bifida, folate supplementation during pregnancy and fortification of food products have become two of the most globally accepted methods of treating and preventing folate deficiency. The basic folate requirement increases 75 to 100% (approximately 300–400 μg per day) in pregnancy because folate has a critical role in the growth and development of the embryo/fetus, especially during early stages of development [13]. It is, therefore, common practice to recommend that women supplement their diet with folate before conception and throughout pregnancy. The prevention of folate deficiency during pregnancy is achieved by consumption of at least 0.4 mg/day of folic acid during the first trimester of pregnancy [14,15]. In light of the recently discovered FRAb that can block folate transport, women positive for these antibodies may need additional supplementation with folinic acid to provide adequate folate to the developing fetus [16,17]. 

## 3. Folate and Fetal Brain Development

The importance of folate during embryonic and fetal brain development has been demonstrated in genetic animal models and dietary manipulations of folate deficiency [18,19]. If either folate transport or folate concentration in circulation is adversely manipulated, embryonic and fetal development is significantly altered. Mouse knockout models of genes such as FOLR1 that encode for folate receptor alpha (FRα) produce lethality in litters along with orbito-facial abnormalities, congenital heart defects and/or neural tube defects [20]. In FOLR1 knockout mouse, these lethalities can be prevented with adequate folinic acid (N5-formyltetrahydrofolate, a reduced form of folate) supplementation. These dramatic results occur because folate transport is lacking in the KO mouse during the early stages of neurulation and in regions where abnormalities arise [21]. In rodent models, folate deficiency causes a decrease in progenitor cells and an increase in apoptosis, and this could lead to infertility or resorption of embryos or fetal malformations [22]. Behavioral deficits are seen in rat pups born to folate-deficient mothers [23] and on methyl donor deficient diet during pregnancy [24]. In a rat model of exposure to rat folate receptor antibodies during pregnancy, resorption of embryos and malformations of the cranio-facial region and the brain were reported [25]. When the antibodies were administered at lower doses, embryos were carried to term with normal appearing pups born. However, these pups showed severe behavioral deficits [23,26]. The behavioral phenotype can be rescued by treatment with folinic acid and dexamethasone prior to antibody exposure [27]. These studies provide strong evidence in support of the pathologic consequences of exposure to FRα antibodies and the protective role of folinic acid.

## 4. Folate and Neonatal Brain Development

**Figure 1:**
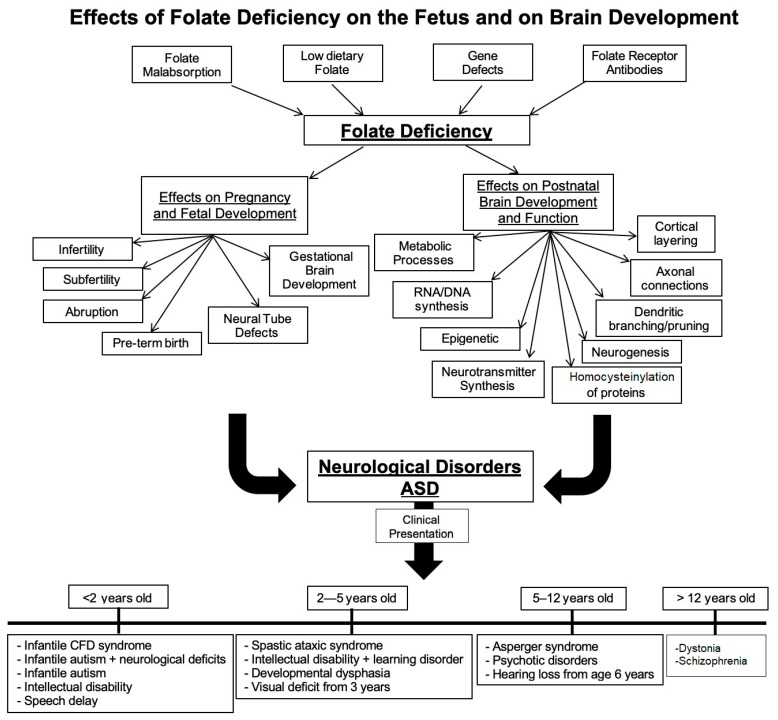
Effects of folate deficiency on the fetus and on brain development. Multiple causes lead to systemic as well as fetal folate deficiency. Folate receptor autoantibodies can block folate transport to the fetus and to the fetal as well as neonatal brain. In addition to folate deficiency, immune-mediated inflammation can contribute to the pathology. This has multipronged effects on brain development and function.

After birth, it is crucial for the offspring to have an adequate amount of folate in their diet. Instead of rapid cell division as embryogenesis calls for, postnatal development requires folate for neural progenitor differentiation as well as proliferation [28]. It has yet to be fully elucidated what the detailed mechanisms of folate action are, but the folate deficiency produced in animal models during early postnatal development illustrates the importance of folate in preventing developmental and cognitive deficits [23,27]. Researchers have also reported changes in neuronal excitability and maintenance that arise with a decrease in brain folate in a rat model [29]. Others have reported an increase in p53 and signs of homocysteine accumulation in the neurons and astrocytes [30]. There was a long-term effect on locomotor function and cognition in these animals. Therefore, folate is necessary for maintenance of neuronal function, as well. Based on this, further investigations into the mechanisms of folate metabolism in neurons and support cells of the brain are necessary. Thus far, folate has been linked to neuronal repair and differentiation after injury, myelin formation and maintenance and neuronal plasticity [30,31,32]. Figure 1 provides a summary of the effects of folate deficiency on fetal and post-natal brain development and the consequent sequelae that contribute to neurologic deficits.

## 5. Folate Receptors: Expression and Function

In humans, there are four genes that code for folate receptors (see Table 1). The most characterized of these receptors is folate receptor alpha (FRα). As extracellular receptors, FRα, FRβ, FRγ and FRδ function as transporters of folate across different target tissues [33,34]. FRα can also act as a transcription factor [33]. Other transporters of folate include the reduced folate carrier (RFC), which requires high local concentrations (micromolar) of biologically active reduced forms of folates, and the proton-coupled folate transporter (PCFT), which can only transport folates and folic acid under acidic conditions and is the primary transporter involved in folate absorption in the gut [35]. 

## 6. FRα Role in Maternofetal Transport of Folate

The high demand for folate during pregnancy requires homeostatic mechanisms to ensure that sufficient folate is provided to the fetus throughout development. As the most characterized receptor in the folate transporter family of proteins, the accepted mechanism of FRα-mediated transport is translocation/endocytosis of the holo receptor subsequent to folate binding [35]. FRα is expressed on all epithelial cells including the choroid plexus. It is highly expressed in the reproductive tissues including the placenta and the fetus. To determine the mechanism of folate transport in the placenta during pregnancy, Yasuda et al. [41] manipulated osmolarity, concentrations of phosphatidylinositol-specific phospholipase C inhibition and concentrations of ^3^H-folic acid *in vitro* culture of human placental brush border membrane vesicles and determined that FRα, RFC and PCFT could transport various forms of folate, but that approximately 60% of folate was binding to FRα. They also noted that the folate requirements of Wistar rats increased across gestation, and expression of the mRNA of the transporters increased as well. 

## 7. FRα Role in Folate Transport to the Brain

FRα is accepted as the main transporter of folate into the brain. However, there have been limitations to studying how FRα transports folate across the blood–brain barrier. A potential mechanism of folate transport across the choroid plexus and into the brain has been described by Grapp et al. [42]. In their experiments using immortalized Z310n rat choroid plexus cells in culture and a mouse model, they determined that transport of folate required shuttling of folates via exosomes from the basolateral side of the choroid plexus to the brain parenchyma of the apical side. Alternative transporters such as RFC and PCFT may only play a role when there is a disruption of FRα expression and transport, and adequate folate concentration is made available locally at the receptor [43]. The shuttling across the epithelial lining of the choroid plexus is a mechanism presumed to be conserved in all tissues that express FRα [44]. 

## 8. Folate Receptor Autoantibodies: Their Role in Disrupting Folate Transport

In some conditions, there is disruption in folate utilization that is not related to a dietary deficiency but is most likely due to a disturbance in the folate’s transport due to genetic or metabolic abnormalities. An emerging culprit of folate transport disruption is folate receptor autoimmune disorder, where autoantibodies against the FRα can interfere with folate transport to the fetus; it has been associated with subfertility, difficulty in conceiving, miscarriage and neural tube defects in the fetus [16,17,45,46]. 

In infants and young children, these antibodies can block folate transport to the brain. Approximately 70% of the children diagnosed with cerebral folate deficiency syndrome or autism spectrum disorder have low CSF folate and respond to folinic acid treatment [47,48]. The majority of the autoantibodies are of the IgG class and, therefore, can readily cross the placenta and affect the fetus. Two distinct types of antibodies have been identified. One binds to FRα at the active site where folate binds and, as a consequence, blocks folate binding (blocking Ab). Another type of antibody binds to an antigenic site not involved in folate binding (binding Ab) but can trigger an immune reaction and inflammation and render the receptor nonfunctional. In most cases, one or both types of antibodies are present [49,50]. Thus, functional blocking of folate transport and inflammation are an integral part of the pathology [44].

## 9. Pathologic Consequences of Folate Receptor Antibodies

The presence of folate receptor autoantibodies can disrupt the transport of folate, and the consequences of decreased folate uptake by cells can impact development of the fetus, especially the central nervous system. There is also a correlation of folate receptor antibodies with neural tube defect pregnancy [16]. In less severe cases, a subset of children born with exposure to maternal FRα autoantibodies *in utero* develop low-functioning autism with or without neurological deficits after birth. Recent studies show significant association of folate receptor autoantibodies with autism spectrum disorder in children [11,51,52].

## 10. Diagnosis of Folate Receptor Autoimmune Disorder

Early indications of cerebral folate deficiency that are potentially due to maternal folate deficiency or folate receptor autoantibodies can be deduced by measuring serum folate and homocysteine and folate receptor autoantibodies in the mother during pregnancy. Other than dietary folate deficiency, folate receptor autoantibodies in the pregnant mother can contribute to fetal folate deficiency. In the latter case, blocking of folate transport across the placenta and antibody-mediated inflammation could contribute to the pathology, as shown in the rat model of exposure to rat folate receptor antibodies during pregnancy [26,27]. In infants, the presence of folate receptor autoantibodies in the blood could provide a mechanism by which folate transport to the brain via the choroid plexus could be blocked, thus leading to cerebral folate deficiency [51,52]. Therefore, determining the presence of folate receptor autoantibodies in the blood of pregnant mothers and children becomes a necessary test to prove or rule out folate receptor autoimmune disorder. 

Methodology for determination of the antibody titer in serum is well-established. Two distinct types of IgG and/or IgM antibodies have been described [50]. These antibodies can be blocking and/or binding antibodies. Both types of antibodies are capable of triggering an immune reaction due to antigen/antibody interaction, leading to local inflammation, and this could interfere with folate transport via the FR protein. Both types of assays can be performed in a laboratory setting as described below.

## 11. Assay for Blocking Antibodies

Blocking autoantibodies to FRα are determined using a functional binding radio assay. Patient’s serum (200 μL) is acidified with 300 μL 0.1 M glycine/HCl pH 2.5/0.5% Triton X-100/10 mM EDTA and added to 12.5 mg charcoal pellets in a separate tube (250 μL of 5% charcoal/1% dextran in 0.1 M Na PO4 pH 7.4/0.5% Triton X-100/10 mM EDTA, spun down and supernatant-aspirated) to remove any endogenous folate, and the pH of the supernatant fluid is neutralized with 40 μL of 1 M dibasic NaPO4 prior to using it in the assay. This assay is performed by adding purified apo human FRα protein (40 ng) to the processed serum and incubating overnight at 4 °C. The next day, ^3^H-folic acid (700 pg) (Moravek) is added and incubated for 20 min at room temperature. Unbound ^3^H folic acid is removed with dextran-coated charcoal (200 μL) and the ^3^H folate bound to FRα determined by counting the sample in a liquid scintillation counter. The reduction in binding of ^3^H-folic acid to the apo human FRα when compared to the negative control serum sample provides a measure of the blocking autoantibody present in the sample [50]. Blocking antibody can be IgG or IgM; the values are expressed as pico moles of ^3^HPGA blocked per ml serum, and the titer can range from >0.2 to 0.5 (low titer), >0.5 to 1.0 (medium titer) or >1.0 (high titer).

## 12. Assay for Binding Antibody

Binding of the IgG autoantibody to folate receptor alpha (FRα) is determined by an ELISA-based method. FRα (1 μg in 100 μL) purified from human milk is added to each well of an ELISA plate to covalently bind the protein to maleic anhydride-coated wells (Thermo Fisher, Waltham, MA, USA). Following blocking of additional sites by treatment with normal goat serum (200 μL) overnight to prevent non-specific binding to the wells, serum samples (4 and 8 μL) (negative control, positive control and patient samples) are added to wells along with 100 μL fresh goat serum and incubated at 4 °C overnight to facilitate binding of autoantibodies to the FRα in the wells. Following washing of the wells to remove unbound proteins, the specific IgG autoantibody bound in each well is detected by incubating with a peroxidase-conjugated, anti-human IgG secondary antibody (1:6000 dilution) (Vector Labs) for 1 h at room temperature. After washing to remove the unbound secondary antibody, the bound peroxidase-conjugated secondary antibody is determined by incubation with ultra TMB (Thermo Fisher) for 10 min. The resultant blue colored reaction is converted to yellow with 100 μL of 1.0 M HCl, and then absorbance is read at 450 nm in an ELISA plate reader. In a second set of wells, known amounts of human IgG captured in protein A-coated plates are used to construct a standard curve [50]. Values are expressed as pico moles of IgG antibody per ml serum and can range from >0.1 to 0.5 (low titer); >0.5 to 2.0 (medium titer) and >2.0 (high titer).

Among other criteria, specific diagnosis of folate receptor autoimmune disorder is confirmed using the above tests. After correcting for background, for the blocking antibody, values of 0.2 pmoles or greater are considered positive and for the binding antibody, 0.1 pmoles or greater are considered positive. Because folate receptor alpha is a peripheral membrane protein, the antibody titer measured in the serum should be considered as excess antibody appearing in the circulation after saturating the membrane-bound antigen. Fluctuations in antibody titer have been reported in the same individual over time and can range from low to medium titer or to undetectable levels. While the reason for these changes in antibody titer are not identified, it is likely that changes in FR antigen on cells, exposure to milk FR antigen in the gut and the specific B-cell population may be contributory factors.

## 13. Treatment of FRα Autoimmune Disorder in ASD 

Among the developmental disorders, ASD is most prevalent and has continued to increase over the past decade. Based on available publications, the WHO reports the worldwide prevalence at 1 in 162 births [53]. In the USA and Canada, the prevalence is reported at 1 in 50, and this rate is predicted to increase over the next few years [54]. While the clinical phenotype of ASD may result from multiple genetic, epigenetic and environmental factors, nutrient deficiencies such as folate can play a significant role. Folate receptor antibodies and cerebral folate deficiency are prevalent in ASD. Treatment of FRα autoimmunity in ASD is based on our previous findings in infantile-onset CFD syndrome and low-functioning autism associated with neurological deficits [11]. In these children, a repeat CSF analysis after three to six months of treatment with folinic acid showed normalization of 5-methyl-tetrahydrofolate levels [11,51]. Supplementation with high-dose dl-folinic acid (Leucovorin) (0.5–2 mg/kg body weight or 0.25–1.0 mg levofolinate) given in 1 or 2 divided daily doses increases 5-methyltetrahydrofolate concentration by more than 100-fold compared to physiological folate concentrations in plasma. Despite the autoantibody-induced blocking of the FRα-pathway to transport folate across the choroid plexus, the significant increase of 5-methyltetrahydrofolate and folinic acid in plasma will enable reduced folate carrier-1 (RFC-1), a high capacity/low affinity transmembrane folate transporter at the blood–brain barrier, to transport sufficient 5-methyltetrahydrofolate and folinic acid to the brain. In this context, it appears important to verify a normal vitamin D status because RFC-1 gene transcription depends on vitamin D availability within microvasculature cells at the blood–brain barrier [40]. 

**Figure 2:**
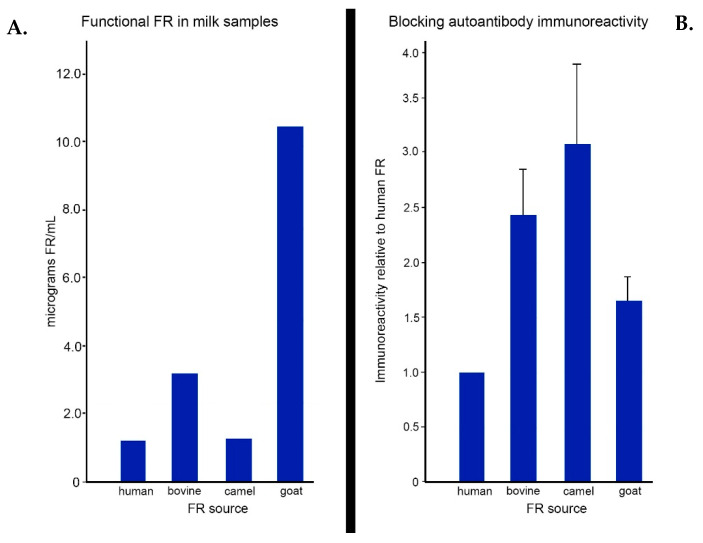
(**A**) Folate receptor concentrations in milk. (**B**) Immune cross-reactivity of blocking autoantibodies against various FR antigens. The blocking assay was performed by testing a known amount of blocking activity in serum samples from 8 different patients with molar equivalent amounts of FR antigens from milk. Blocking activity was determined as pico moles of ^3^HPGA blocked and was compared to blocking in human milk antigen.

Another important therapeutic intervention represents a diet strictly free of animal-derived milk or milk products, which can be replaced by other vegetal milk products (for example soya-, almond- and rice-based and coconut milk). Although many previous studies on a casein/gluten-free diet have been conducted, there has been no final evidence yet to consider these dietary treatments as beneficial in the management of ASD. Many studies have been conducted for a maximum of only 3 months, although some studies on a small number of patients were conducted over 1 to 2 years and indicated that part of the core symptoms of autism had improved [55,56,57,58]. The conclusion was that a casein/gluten-free diet should be tried for at least 6 months to see a positive response in a subset of the ASD population. One suggested hypothesis was that opioid peptides derived from milk casein contributed to the pathogenesis of autism [59]. Because bovine milk contains a soluble form of the FRα protein with 91% homology with human FRα, we examined the binding properties of human FRα autoantibodies with different FRα antigens isolated from human placenta; human milk; and bovine, goat and camel milk. The highest cross-reactivity of the autoantibody was found for soluble FRα protein from bovine and camel milk (Figure 2). To determine if FRα in the milk consumed contributed to the autoimmune disorder, we studied the effects of a milk-free diet in children positive for the FRα antibody. Patients with infantile CFD syndrome associated with FRα antibodies were randomized to receive either a cow’s milk-free diet or a normal, milk-containing diet. Among children on a normal diet, FRα antibodies increased from baseline toward higher titers during 6–12 months of evaluation. However, the children receiving a milk-free diet showed a significant drop in FRα antibody titers after 3–6 months that rose again after re-introduction of bovine milk. These studies confirmed down-regulation of the FRα antibodies following a strict animal milk-free diet [55]. In this group of patients with infantile CFD syndrome, a number also suffered from low-functioning autism with neurological deficits and showed a clinical response after a milk-free diet. These findings suggest that in predisposed individuals, the soluble FRα antigen derived from bovine and other animal-derived milk products acts as the antigen that triggers a gut immune response with the formation of specific B-cell clones that produce autoantibodies that enter the circulation, cross-reacts with the human FRα anchored to the choroid plexus and blocks folate transport from the circulation into the CSF [44,60]. Thyroid dysfunction is common in children with ASD. Even though FRα expression in the thyroid gland is decreased in older children and adults, it is highly expressed in the fetal and neonatal thyroid, and FRα antibodies can affect development of the thyroid gland [61]. A preferred strategy for individuals with autism spectrum disorder is to take a serum sample for determination of FRα autoantibodies after exposure to milk products for about 2–3 weeks. After this diagnostic blood test, autistic children can be placed on an animal milk-free diet. As soon as the FRα autoantibodies test positive, a milk-free dietary intervention can be continued along with high oral doses of folinic acid. Other treatment strategies to reduce FRα autoantibodies may be immunosuppression using steroids or intravenously administered immunoglobulins, but these therapeutic options should be reserved for emergency situations such as refractory epileptic seizures or dramatic movement disorders such as dystonia, choreoathetosis or ballism.

**Figure 3:**
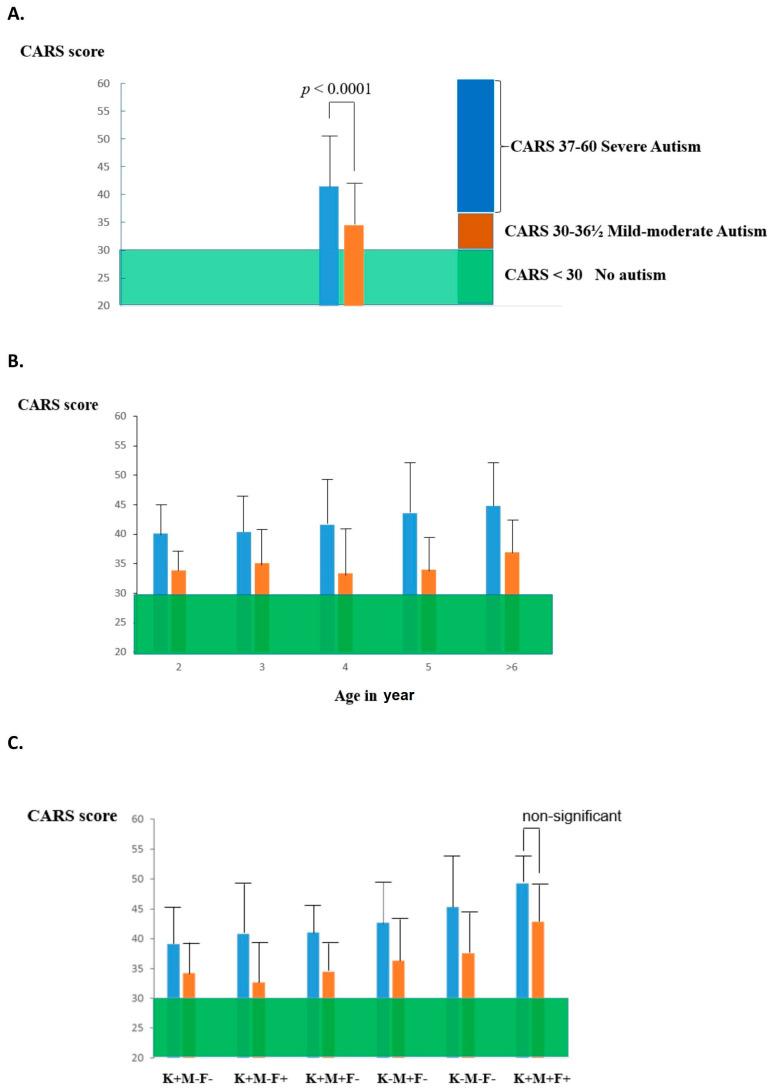
(**A**). Compared to untreated autistic patients (*n* = 84) whose CARS remained unchanged, a group treated with folinic acid and with their nutritional deficits corrected showed a decrease in baseline CARS score from severe (*n* = 82; CARS mean ± SD: 41.34 ± 6.47) to moderate or mild autism (mean ± SD: 34.35 ± 6.25; paired *t*-test *p* < 0.0001). (**B**). As a function of age, the baseline CARS (blue bars) increased slowly with advancing age, while the CARS after a 2-year treatment period (orange bars) diminished significantly for all age subgroups. The increase of baseline CARS with advancing age will adversely influence the final outcome for older age groups, particularly above 6 years. (**C**). This graph represents the outcome of treatment as a function of the particular FRα antibody profile in the child (K), mother (M) and father (F). The presence of maternal FRα antibodies or presence of antibodies in both parents will negatively affect the treatment outcome (adapted from [51]).

Treatment with high-dose folinic acid in a subgroup of ASD children positive for FRα autoimmunity, i.e., low-functioning autism with neurological deficits, showed clinical improvement of core autistic symptoms and normalization of previously lowered CSF 5-methyltetrahydrofolate [51]. A double-blind, placebo-controlled study conducted among children with ASD without additional neurologic deficits showed significant improvements in verbal scores in subjects positive for FRAb following treatment with folinic acid [48]. A recent, self-controlled clinical trial was conducted among children with low-functioning autism without additional neurological complications. In these, a high, 76% prevalence of FRα antibodies was found. These children also had multiple nutrient deficiencies attributed to selective eating habits and malnutrition. Combined correction of deficient nutrients and high dose folinic acid administration resulted in an overall significant recovery from severe autism to mild–moderate autism (Figure 3A). Comparison of the Childhood Autism Rating Scale (CARS) after 2 years of treatment (folinic acid supplementation and correction of abnormal nutrient values) with the CARS at baseline showed better outcomes for children having negative or low FRα antibody titers of the blocking type, up to 0.44 pmol FRα blocked/mL serum, versus the group whose FRα antibody titers were above 0.44. The baseline CARS score increased as a function of the age at which treatment was initiated. The outcome became poorer for the older subgroup of treated autistic children (Figure 3B). This outcome may be further compounded by the presence of maternal and paternal autoantibodies and embryonic exposure to these. Preliminary data suggested that in the event of maternal or parental FRα autoantibodies, the child´s outcome after treatment was also less favorable (Figure 3C). 

Compared to infantile-onset CFD syndrome where FRα antibody testing remained negative in the parents, testing of the parents of children with autism revealed a prevalence of 34% in mothers and 29% in fathers versus 3% in healthy controls [51]. Another study also confirmed an equal prevalence of FRα autoimmunity in children with autism (76%) and even higher autoantibody prevalence in their unaffected siblings (75%), fathers (69%) and mothers (59%) [62]. The appearance of these antibodies may have a familial heritable origin, but the risk of developing ASD is likely influenced by other unknown factors because some siblings positive for these antibodies have been asymptomatic. Two of the suspected determinant factors for the development of autism are the appearance of antibodies at a critical stage of neurodevelopmental processes during the first 18 months of life and fetal exposure to maternal antibodies.

**Figure 4:**
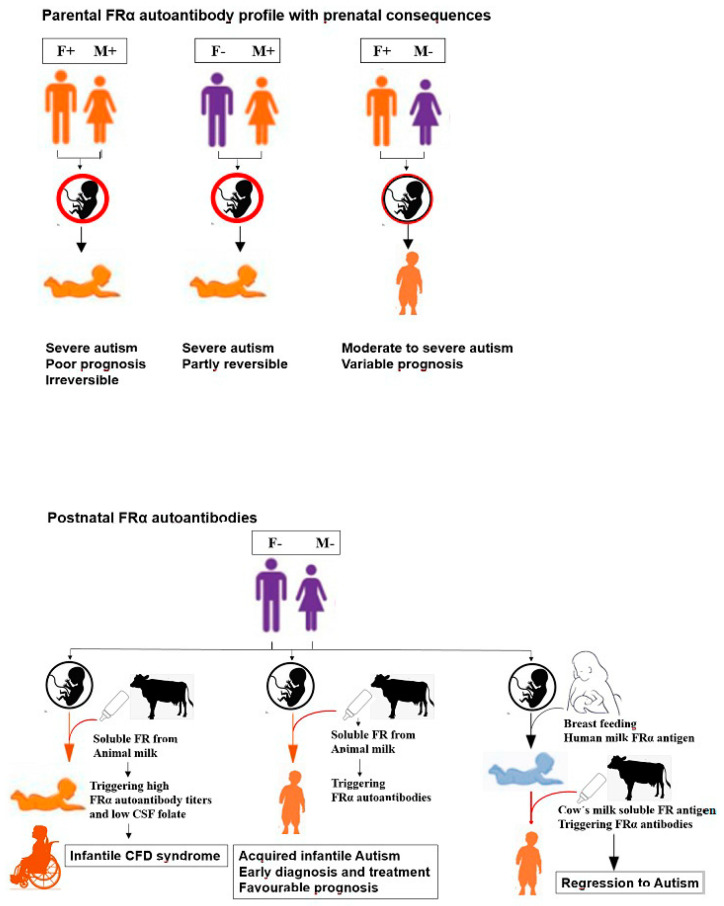
The significance of parental antibody status on developmental outcomes in offspring (top panel) and postnatal antibody development in offspring (bottom panel).

The outcome after folinic acid treatment of autism associated with FRα autoimmunity appears to be influenced by several factors such as the level of FRα antibody titer and age at which treatment was initiated as well as the FRα antibody profile amongst parents. In our studies on the treatment outcome after folinic acid therapy for two years we only included the group of children with infantile-onset autism in whom genetic abnormalities had been excluded because genetic defects might constitute a bias to statistical assessment regarding the influence of FRα autoimmunity (Figure 4). 

## 14. Treatment of FR Autoimmune Disorder in Pregnancy 

FRα autoimmunity has been associated with a high risk of neural tube defects (NTD) and other congenital malformations in offspring [16,17]. This has been confirmed by other independent studies [63,64]. Even though the incidence of NTD is high in the Irish population, evaluation of FRα autoantibodies in this population has failed to show a statistically significant correlation with NTD pregnancies [65]. However, the study showed a higher prevalence (35–40%) of FR antibodies in the mothers and both male and female controls. Because FRα antibodies were not assessed in the fathers of the children with NTD and only in unrelated males of the control group, the contribution of the possibility of both parents being positive for FRα antibodies to the NTD outcome cannot be ruled out. Parental studies in ASD have shown both maternal and paternal influences on the incidence and severity of ASD outcome [51]. The folic acid fortification of foods has reduced the prevalence of NTD by 30–50 percent. However, for women having a normal folate status but testing positive for FRα autoantibodies, we suspect that even the addition of a daily dose of 400–800 µg or 1000 µg for twin pregnancies may not prevent NTD or congenital malformations due to the persistence of embryonic and fetal folate deficiency in the presence of FRα antibodies. 

One case report described a woman who, upon follow-up after three pregnancies, was found to have high titers of serum FRα autoantibodies of the blocking and binding types. She had previously had two miscarriages and a third pregnancy with monozygotic twins, during which she took 1 mg folic acid per day. However, one twin was reduced at 12 weeks because of an encephalocele, and the pregnancy was terminated because the other twin had hypoplastic left-heart syndrome and choroid plexus cysts. At this time, extensive genetic testing did not reveal any abnormalities. After finding FRα autoantibodies, treatment with a milk-free diet was able to reduce FRα antibody titers, but a fourth pregnancy by IVF also resulted in a miscarriage after 5 months. It was only after continuation of the milk-free diet combined with 4 mg folic acid, 2.5 mg leucovorin and 5 mg prednisone that FRα antibody titers fell to undetectable levels, and a fifth pregnancy was carried to term and resulted in the birth of a healthy baby boy [17]. FRα autoantibodies are significantly associated with subfertility and preterm birth [45,46], and preterm babies have a higher prevalence of ASD [66]. Therefore, testing for FRα antibodies in women of child-bearing age may help in preventing some of these disorders by early intervention.

## 15. Prevention of ASD and Related Complications Due to FR Antibodies 

Prevention of ASD has not been reported yet. However, it seems extremely important to diagnose ASD at the earliest age possible in order to be able to perform further evaluation including testing for serum FRα autoantibodies. We believe that as soon as ASD is strongly suspected and FRα antibodies identified as early as possible prior to the age of three years, the outcome following prompt treatment with high-dose folinic acid in combination with an animal milk-free diet will have a favorable outcome if maternal FRα antibodies or the presence of antibodies in both parents were negative [51].

Currently, FRα antibody testing is only performed after ASD is suspected or has been diagnosed. However, this procedure postpones treatment and causes a significant delay, affecting prognosis unfavorably. Therefore, the early screening of autism at 18 or 24 months using the Modified Checklist for Autism in Toddlers (M-CHAT test) or other instruments can be used by health workers, although there is lack of optimal sensitivity and specificity for ASD when using these tests at an early age.

Another option would be to perform the screening test for FRα antibodies at an early age between 12 and 18 months, particularly among those children suspected to manifest one or more autistic signs or symptoms. The children testing positive for FRα antibodies could be placed on an animal milk-free diet and receive folinic acid supplementation with a mandatory follow-up of these children.

## 16. Clinical Significance of the Findings

Since the discovery of folic acid more than a century ago, the hematologic consequences of its deficiency and its role in DNA synthesis and treatment of megaloblastic anemia has been well defined. Emerging research aims to define its role in methylation reactions, epigenetic regulation of gene expression, reproductive function, pregnancy and fetal development. It is becoming evidently clear that folate plays a major role not only in fetal brain development but also in post-natal development and refinement of functional integration of the mature brain. Clinical improvement seen in response to folinic acid treatment in ASD, schizophrenia, depression and dementia attests to the role of folate in metabolic regulation of brain function, potentially by regulating the expression and processing of neurotransmitters. While conventional thinking would associate disruption of folate metabolism with nutritional folate deficiency and gene defects of folate pathways, the identification of folate receptor autoantibodies contributing to fetal and cerebral folate deficiency has thrown a monkey wrench into our current thinking regarding folate transport into the brain and its role in regulating brain function. Therefore, clinical recognition of cerebral folate deficiency is critical to our understanding of neuro–developmental as well as neuro–psychiatric disorders.

To prevent fetal folate deficiency, specific guidelines for treatment of future parents testing positive for FRα antibodies should consider the time frame and dose for folinic acid supplementation prior to conception and for mothers, the folinic acid dose to be used during pregnancy. It will be extremely important to assess optimal dosage in order to provide sufficient folate supplementation but avoid excess dosing, especially since data are lacking on the safety profile of high-dose folinic acid administered throughout pregnancy. Based on the safety profile of high-dose folinic acid used in the treatment of CFD and ASD, one may speculate that a daily dose of 5–10 mg may be in the safe range. The future availability of levofolinate could reduce this dose by half. For favorable outcomes in CFD and ASD, early testing for FRAb and treatment with folinic acid could potentially prevent the development of neurologic deficits.

## 17. Concluding Remarks

Decades of research into neural tube defect pregnancies have only managed to reduce their incidence through folic acid supplementation, but not prevent them altogether. ASD incidence, on the other hand, has continued to rise with no definitive contributing cause identified. Both public and private funding agencies have poured a major share of available funds toward identifying gene defects and genomic polymorphisms to no avail. An enormous sum of money has been expended in developing gene deletion mouse and rat models to identify the autism gene(s). It is now clear that ASD is not a congenital genetic disorder and does not follow Mendelian inheritance. Therefore, the answer to the pathogenesis of ASD must lie in epigenetic and environmental factors that broadly affect gene expression. Folate plays a pivotal role in DNA/RNA synthesis, methylation and epigenetic control of gene expression, and therefore, decreased folate availability during critical stages of development, albeit by the presence of FRAb-blocking folate transport as well as triggering inflammation, may play a significant role in the pathology of ASD.

## Figures and Tables

**Table 1:** Summary of folate transporters.

Protein	Gene	Chromosome	GPI Anchor?	Localization	Cofactors?	Refs.
FRα	FOLR1	11q13.3	Yes	Liver, kidney, uterus, placenta, choroid plexus, retinal pigment epithelium	LRP2	[33,35,36,37,38]
FRβ	FOLR2	11q13.4	Yes	Placenta, spleen, bone marrow, thymus, macrophages	NA	[33,35,36,37,38,39]
FRγ	FOLR3	11q13.4	NA	Secretory granules of neutrophil granulocytes	NA	[33,35,36,37,38,39]
FRδ	FOLR4	11q14	Yes	Oocytes	NA	[33,35,36,37,38,39]
RFC	SLC19A1	21q22.3	No	Liver, kidney, placenta, choroid plexus, intestinal tract	Vitamin D, thiamine pyrophosphate	[34,40]
PCFT	SLC46A1	17q11.2	No	Liver, kidney, choroid plexus, placenta, intestinal epithelium, human tumors	Proton gradient	[34,40]

## Data Availability

Not Applicable.
